# Growth of epitaxial silicon nanowires on a Si substrate by a metal-catalyst-free process

**DOI:** 10.1038/srep30608

**Published:** 2016-07-28

**Authors:** Takeshi Ishiyama, Shuhei Nakagawa, Toshiki Wakamatsu

**Affiliations:** 1 Department of Electrical and Electronic Information Engineering, Toyohashi University of Technology, 1-1 Hibarigaoka, Tempaku-cho, Toyohashi, Aichi 441-8580, Japan

## Abstract

The growth of epitaxial Si nanowires by a metal-catalyst-free process has been investigated as an alternative to the more common metal-catalyzed vapor–liquid–solid process. The well-aligned Si nanowires are successfully grown on a (111)-oriented Si substrate without any metal catalysts by a thermal treatment using silicon sulfide as a Si source at approximately 1200 °C. The needle-shaped Si nanowires, which have a core–shell structure that consists of a single-crystalline Si core along the <111> direction consistent with the substrate direction and a surface coating of silicon oxide, are grown by a metal-catalyst-free process. In this process, the silicon sulfide in the liquid phase facilitates the nucleation and nanowire growth. In contrast, oxygen-rich nanowires that consist of crystalline Si at the tip and lumpy silicon oxide on the body are observed in a sample grown at 1300 °C, which disturbs the epitaxial growth of Si nanowires.

Semiconductor nanostructures have been studied widely because of their unique electrical and optical properties. In particular, one-dimensional structures, such as nanotubes, nanorods, nanowires, and nanoneedles, have attracted a great deal of attention as promising candidates for new optoelectronic devices^1^,^2^,^3^. Epitaxial Si nanowires vertically grown on a Si substrate are particularly expected to be applied as new integrated devices because they are compatible with existing complementary metal-oxide-semiconductor (CMOS) technology; some devices based on Si nanowires as building blocks have already been demonstrated^4^,^5^,^6^,^7^. Silicon nanowires with well controlled electrical and optical properties are needed to fabricate the devices, so the growth of epitaxial nanowires with good crystallinity is very important for obtaining nanowire building blocks. Therefore, the properties of silicon nanowires prepared by various methods have been studied intensively for obtaining high-quality nanowires^8^,^9^,^10^,^11^,^12^,^13^. Epitaxial growth of well-aligned nanowires on a Si substrate is required for the application of Si nanowires as a device platform in electronic and photonic devices. The vapor–liquid–solid (VLS) method using a metal catalyst is one of the most prevalent approaches for the growth of Si nanowires because it has some advantages, including a simple method and low cost compared to other methods such as chemical vapor deposition, solution phase synthesis, and photolithography techniques. In addition, the VLS method enables the growth of epitaxial nanowires on a single-crystal substrate. In the conventional VLS method, gold (Au) is typically used as a catalyst because Au and Si easily form alloy droplets at a relatively low temperature. Gaseous Si is preferentially absorbed into the alloy droplets on a Si substrate. When the Si is supersaturated in the alloy droplet, the crystallization of Si starts at the liquid–solid interface on the Si substrate. Subsequently, the Si, which is continuously absorbed into the alloy droplet, is added to the growth of the nanowire. This reaction results in the epitaxial growth of the Si nanowire. Finally, the tip of the Si nanowire grown by the VLS method using a metal catalyst terminates with a metal sphere, which is a solidified droplet; the diameter of the nanowire depends on the size of the metal sphere on the tip^1^,^14^. However, the presence of a metal source contaminates the nanowire with metal impurities. The metal impurities produce energy levels at the bandgap and degrade the electrical and optical properties^15^. There are a number of reports on the incorporations of metal catalysts into nanowires during VLS growth and the influence of the incorporation on the basic properties^15^,^16^,^17^,^18^,^19^,^20^. These investigations are very valuable for understanding the influence of metal contamination on the electrical and optical properties, and to achieve nanodevices with a high performance^4^,^21^,^22^,^23^,^24^,^25^,^26^.

New epitaxial growth methods that do not require any metal catalysts have been investigated as a replacement of the conventional VLS method to prevent metal contamination during Si nanowire growth. There have been several reports on Si nanowires synthesized by laser ablation using mixtures of Si and silicon oxide as the source^27^,^28^,^29^,^30^. Although these methods do not require any metal catalysts, the product is an aggregate of bent nanowires. Crystalline Si nanowires with the axes parallel to the <111> direction were synthesized by heating a mixture of Si and silicon oxide at high temperature^31^. However, silica nanospheres and wire-like nanosphere agglomerates were also synthesized simultaneously, and the crystalline nanowires were a small proportion of the products. The carbothermal method, which is a thermal evaporation method, also does not require the use of metal catalysts for the synthesis of Si nanowires^32^,^33^,^34^. Although the method can be used for the synthesis of nanowires on a Si substrate, the nanowires exhibit bends and kinks. Recently, the metal-free growth of single crystal Si nanowires on Si substrate has been reported^35^,^36^. In these methods, Si substrates covered with silicon oxide are used instead of substrates covered with metal catalyst and the growth process is based on oxide-assisted growth. The growth directions of the nanowires are mainly parallel to the <110> direction, but the product is a sponge-like web of dense nanowires. A summary of the synthetic conditions used for the growth of Si nanowires using various methods without any metal catalysts is presented in Table 1. Thus, the development of the synthesis of single-crystalline Si nanowires on a Si substrate without using any catalytic metal is progressing steadily. However, the epitaxial growth of well-aligned Si nanowires on a Si substrate by a metal-catalyst-free process has not been achieved yet.

Previously, the growth of Si nanowires by a VLS method using both sulfur and catalytic Au has been reported^37^. In this method, sulfur produces vapor-phase Si sulfides by etching the Si substrate and silicon sulfide vapor is used as the vapor-phase source instead of SiH_4_, which is generally the source gas of Si in the VLS process. In contrast, we found that well-aligned Si nanowires can also be grown on a Si substrate without using any catalytic metals by using sulfur^38^. However, the crystallinity of the nanowires was not clear and a lot of nanowires with bends and kinks were observed in addition to straight and smooth nanowires. In this paper, we report the growth of epitaxial Si nanowires on a Si substrate by a metal-catalyst-free process. The epitaxial nanowires are grown uniformly on a (111)-oriented Si substrate by using sulfur and no catalytic metal. The nanowires grown by the metal-catalyst-free process are single-crystalline Si along the <111> direction and the surface is coated with silicon oxide. Many Si nanowires with high oxygen content are grown at higher reaction temperatures, which disturbs the growth of the epitaxial nanowires. The metal-free nanowire contains sulfur atoms that are a constituent of the precursor though the metal-catalyst-free process excludes metal contamination of the nanowire. Therefore, the growth of high-quality nanowire has not been achieved yet because sulfur atoms also form levels in the Si bandgap. The management of sulfur contamination in nanowires is also required because it is difficult to exclude sulfur incorporation. The determination of sulfur distribution and the management of sulfur contamination in the nanowire are issues to be addressed in the future.

## Results and Discussion

**Figure 1:**
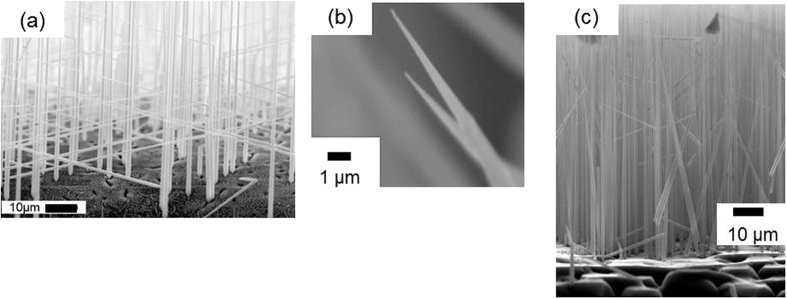
(**a**)SEM image of Si nanowires grown on (111)-oriented Si substrate by the metal-catalyst-free process (**b**) image of the tips, and (**c**) side view of the nanowires.

**Figure 2:**
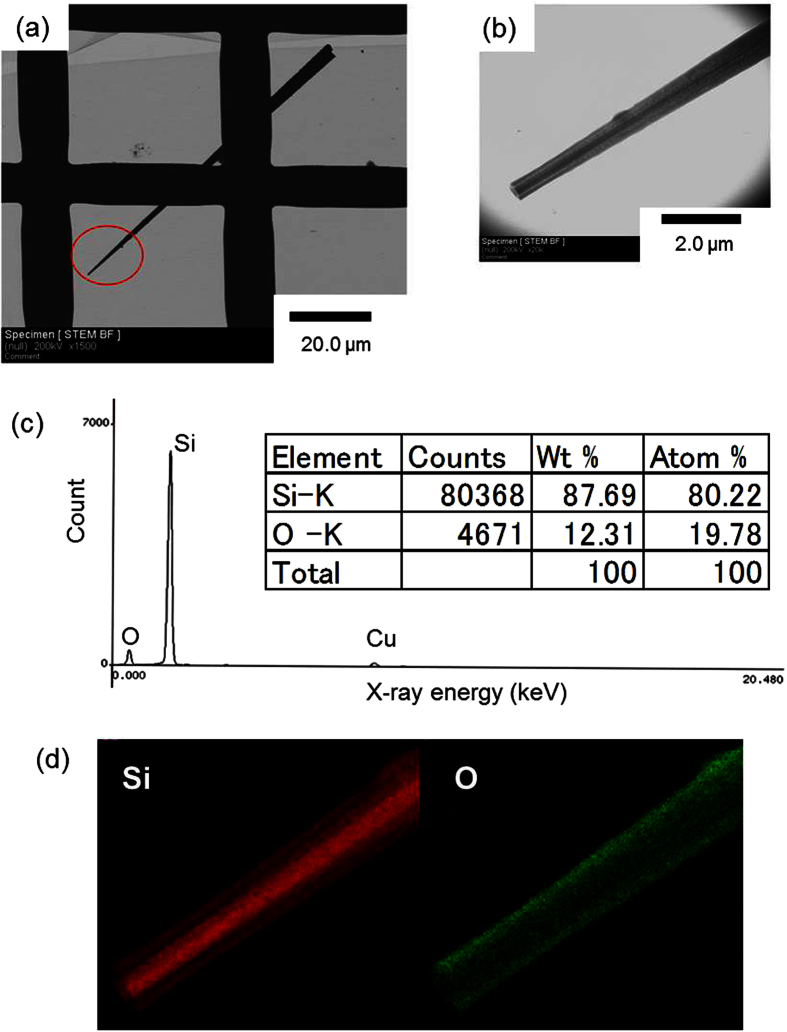
(**a**) TEM image of a Si nanowire grown by the metal-catalyst-free process, (**b**) image of the tip section of the nanowire denoted by the circle in (**a**) and (**c**) EDX spectra of the tip section collected 150 nm from the tip. The peak for Cu is from the grid for TEM. (**d**) Scanning TEM elemental maps of Si (red) and O (green) concentrations in the nanowire.

**Figure 3:**
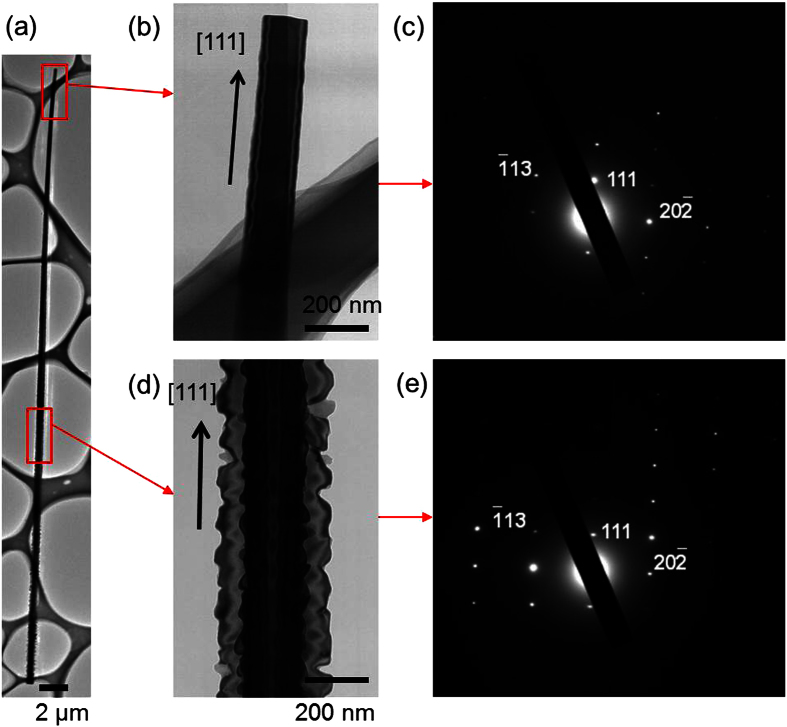
TEM images of a Si nanowire grown by the metal-catalyst-free process, and the electron diffraction patterns. (**a**) Whole image of the Si nanowire, (**b**) an enlarged view of the tip of the nanowire, and (**c**) the electron diffraction pattern recorded along the [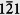] zone axis from the tip of the nanowire. (**d**) The enlarged view and (**e**) the electron diffraction pattern of the middle section of the nanowire.

A simple thermal treatment, which is described in the Methods section, enables the epitaxial growth of well-aligned nanowires on a Si substrate by a metal-catalyst-free process. Typical scanning electron microscopy (SEM) images of Si nanowires grown using this method are shown in Fig. 1. Aligned and uniform Si nanowires can be clearly seen. The nanowires with sharp tips are different from those grown by the conventional metal-catalyzed VLS process. The needle-shaped nanowires are aligned vertically to the (111)-oriented substrate, as shown in Fig. 1(c). Next, we verified that Si nanowires are also grown by the method using sulfur and catalytic Au, as reported in ref. 37. Nanowires with spherical Au tips that are the same as those grown by the conventional metal-catalyzed VLS process are grown on the Si substrate. The elemental mapping shows that sulfur is localized at the spherical tip in addition to Au. The tips of the nanowires grown using the Au catalyst terminate as spheres because of the solidification of alloy droplets of Au and Si. In contrast, without a metal catalyst the nanowires grow in the shape of needles and there are no spheres at the tips, as shown in Fig. 2. Energy dispersive X-ray fluorescence (EDX) analysis of the tip section confirmed that the nanowires are silicon and contain a small amount of oxygen. The EDX spectrum is collected at two points with 2 nm diameter using scanning transmission electron microscope (STEM). The point at the tip is 150 nm from the tip and the point at the middle is 40 μm from the tip. Both points are at the center of the crosswise direction. There are no peaks for metals detected, except for a peak for Cu from the grid used as a sample holder. The elemental composition is 80 at% Si and 20 at% oxygen at the tip section, and 90 at% Si and 10 at% oxygen at the middle section. Both the electron diffraction patterns of the tip and the middle sections of the nanowire correspond to that of single-crystalline Si and the growth direction is consistent with the <111> direction, as shown in Fig. 3. TEM images reveal that the nanowire is a core–shell structure and the elemental mapping shows a localized Si core and an O shell. Therefore, the nanowire consists of a single-crystalline Si core and a surface coating of silicon oxide. Thus, the well-aligned Si nanowires are epitaxially grown on a Si substrate by a metal-catalyst-free process. However, the distribution of the diameter and the length of the nanowires grown by the metal-catalyst-free process are not yet comparable to the uniform nanowires grown by a well-controlled metal catalyzed VLS method. The average length of the metal-catalyst-free nanowires is 85 ± 29 μm with a diameter of 260 ± 140 nm at the tip, which is 500 nm from the tip, 690 ± 300 nm at the middle, which is at the center of the length direction, and 1500 ± 680 nm at the bottom, which is 500 nm from the bottom. The diameters of the nanowires grown by the metal-catalyzed VLS method depend on the size of the alloy droplets on the Si substrate. Modifying the size of the alloy droplets by selecting suitable catalyst materials, catalyst/reactant compositions, and reaction conditions is a common technique used to control the length and diameter of the nanowires. The application of these steps to the metal-catalyst-free method, which uses silicon sulfides produced by a reaction between Si and sulfur as the melted catalyst, is probably useful for controlling the length and diameter of the nanowires. Moreover, a size-control template, such as an anodic aluminum oxide (AAO) membrane, which is widely used as a template for the formation of one-dimensional nanostructures^8^,^39^, can be used to control the diameter and length of the nanowires grown by our method. The tapering degree, defined as *σ* = (*d*_*b*_ *−* *d*_*t*_)*/2L* (*d*_*t*_: the tip diameter, *d*_*b*_: the bottom diameter, and *L*: length)^40^, is estimated to be approximately *σ *= 7.3 × 10^−3^ using the average values. This tapering degree for the metal-free nanowire is comparable with those in refs 40 and 41. The tapering of nanowires grown by the VLS process is caused by Si deposition on the nanowire sidewalls, which is a vapor–solid (VS) growth mechanism^40^,^41^; as such, radial growth in the metal-catalyst-free process also occurred by a VS process of Si deposition from vapor-phase Si sulfide. The tapering degree depends on the process conditions, such as the source gas, flow rate, and pressure. Tapering of the nanowires can be inhibited by adjusting the process conditions. Moreover, utilizing an AAO membrane, which provides a straight size-control template, also ensures the growth of straight nanowires. The growth of well-ordered nanowires using our method is an issue that will be addressed in the future. The metal-free nanowires contain sulfur atoms, which are a constituent of the precursor. Those sulfur atoms form deep levels or double-donor states in the Si bandgap^42^,^43^. The determination of sulfur distribution, which requires atom probe tomography (APT) or secondary ion mass spectrometry (SIMS), and the management of sulfur contamination in the nanowire are also important issues to be addressed in the future. Conventional techniques for the management of metal contamination in bulk semiconductors, such as the gettering effect using another impurity atom, the passivation effect using hydrogen atoms^44^, or the diffusion of sulfur into a substrate by post-annealing, are promising solutions to render sulfur contamination harmless. In contrast, nanowires grown by the common metal-catalyzed VLS method terminate with a metal sphere, which acts as a metal diffusion source through subsequent processes during device fabrication. Therefore, the application of these techniques to nanowires with a metal diffusion source is invalid because metal impurities are continuously diffused from the source at the tip into the nanowire.

We were naturally interested in the epitaxial growth process of nanowires without the use of metal catalyst. In the method using sulfur and catalytic Au, nanowires are grown at 1230 °C, and the source gas in this growth is vapor-phase Si sulfides, which are produced by etching the Si substrate at a higher temperature than the sublimation point of SiS (940 °C) and the boiling point of SiS_2_ (in the 1100–1200 °C range)^37^. In contrast, the growth of epitaxial nanowires by the metal-catalyst-free process is confirmed in samples annealed above 1150 °C, but the growth of the nanowires is not observed in a sample annealed at 1000 °C. The surface morphology of a sample in which a Si substrate sealed with sulfur powder in a quartz capsule is heated at 1000 °C for 30 min was rough because of the etching by sulfur. Namely, silicon sulfide vapor as the source gas is already generated at 1000 °C although nanowires do not grow on the substrate. This suggests that the growth of the nanowires by the metal-catalyst-free process is not caused by the VS process, and a melted catalyst absorbing the silicon sulfide in the vapor-phase is necessary for the epitaxial growth. Thus, the melted catalyst is probably formed at a temperature range from 1000 to 1150 °C, which coincides with the melting point of SiS_2_ at 1090 °C. Therefore, the liquid-phase SiS_2_ is the most likely candidate for the melted catalyst in the metal-catalyst-free process. The metal-catalyst-free growth can probably be attributed to the silicon sulfide acting as both the vapor-phase source and the melted catalyst for VLS growth. The tips of the nanowires grown by the metal-catalyst-free process are like needles and they do not terminate with a sphere of residual catalyst, in contrast with the spherical tip of nanowires grown by the metal-catalyzed VLS method. We expect that most of the residual sulfur that is a constituent of the catalyst at the tip evaporates as silicon sulfide vapor when the temperature decreases after the heat treatment, because silicon sulfide vapor is generated by the reaction between Si and S at temperatures higher than at least 1000 °C, as mentioned above.

**Figure 4:**
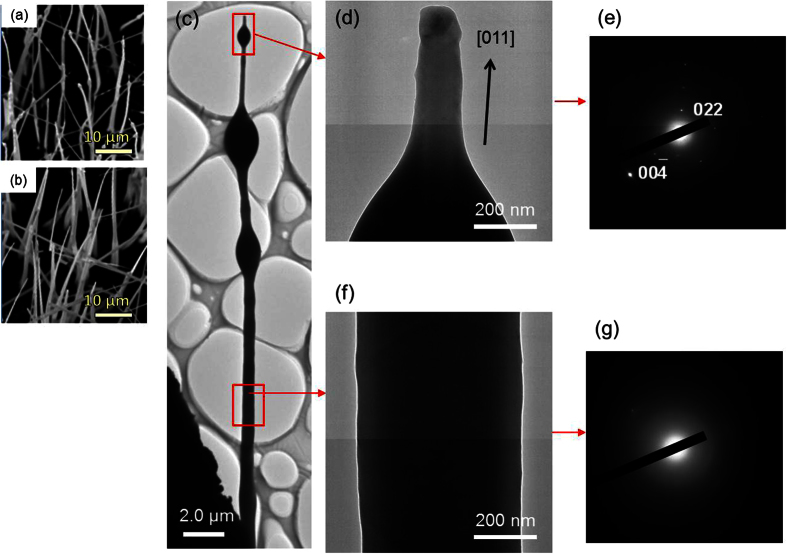
SEM images of nanowires grown at (**a**) 1250 °C and (**b**) 1300 °C. (**c–g**) TEM images of a bent nanowire with lumps, and the electron diffraction patterns; (**c**) whole image of the nanowire, (**d**) an enlarged view of the tip of the nanowire, and (**e**) the electron diffraction pattern recorded along the [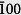] zone axis from the tip of the nanowire. (**f**) The enlarged view and (**g**) the electron diffraction pattern of the middle section of the nanowire.

**Figure 5:**
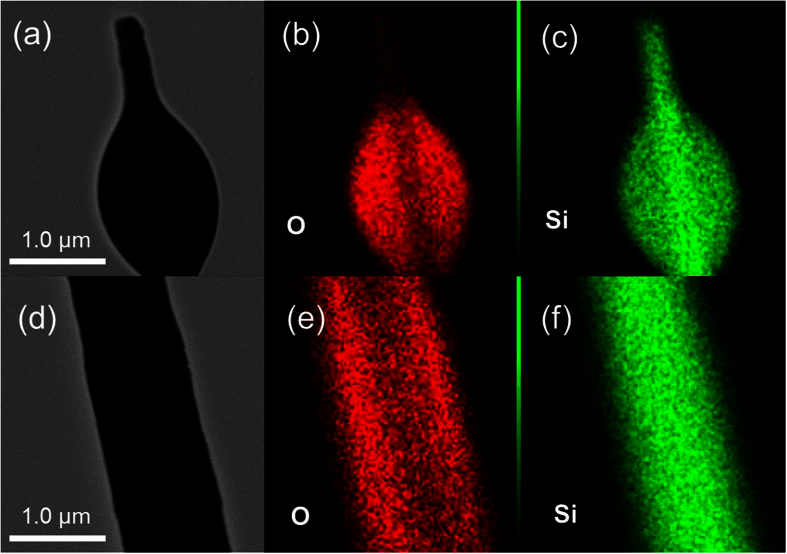
TEM images and the scanning TEM elemental maps of Si (green) and O (red) concentrations in the bent nanowire. (**a–c**) the tip and (**d–f**) the middle section of the nanowire.

Moreover, the number of bent nanowires, which are silicon oxide, increases with increasing growth temperature in the range from 1250 to 1300 °C, as shown in Fig. 4. This suggests that the epitaxial growth of Si nanowires by the metal-catalyst-free process becomes gradually more difficult at temperatures higher than the melting point of SiS_2_. The portion of liquid SiS_2_ decreases with increasing temperature and the number of crystalline Si nanowires also decreases. As a result, the growth of bent nanowires through another process is dominant at the higher temperature. Oxygen-rich nanowires are likely to grow by oxide-assisted growth^29^,^30^. TEM images of the bent nanowires with lumps grown at 1300 °C are also shown in Fig. 4. The electron diffraction pattern shows that the tip section of the bent nanowire is along the <110> direction, but the crystallinity of the middle section of the nanowire could not be identified because it is too thick to measure the diffraction pattern. The distribution of Si is throughout the nanowire and oxygen is relatively localized at the shell and the lump, as shown in Fig. 5. Therefore, the tip section of the bent nanowire is crystalline Si and the body with the lumps is silicon oxide. The tip section is probably grown from a silicon oxide lump by oxide-assisted growth. The large amount of oxygen incorporated into the nanowires originates from residual oxygen in the quartz capsule evacuated to 20 Pa. A larger number of bent nanowires with high oxygen contents were obtained when the quartz capsule was evacuated to approximately 30 Pa, as shown in Supplementary Fig. S1. Epitaxial nanowires were not observed in the sample grown under 30 Pa. An oxygen-rich nanowire is predominantly grown in an atmosphere containing a larger amount of oxygen, which disturbs the epitaxial growth of the Si nanowires. Thus, the growth process of these oxygen-rich nanowires is different from the metal-catalyst-free process. In the capsule heated at approximately 1300 °C, almost all Si sulfides produced by the etching are in the vapor-phase, so there is a very small amount of liquid-phase SiS_2_ melted catalyst, which disturbs the growth of the epitaxial nanowires. This suggests that the liquid-phase SiS_2_ functions as the catalyst for the growth of epitaxial Si nanowires.

## Conclusions

We have investigated the growth of epitaxial Si nanowires by a metal-catalyst-free process. The well-aligned Si nanowires can be epitaxially grown on a (111)-oriented Si substrate by using sulfur and no catalytic metal. The needle-like Si nanowires are grown along the <111> direction and consist of a single-crystalline Si core and a silicon oxide shell. In this synthesis technique, the Si nanowires are probably grown by the VLS process assisted by sulfur. It is suggested that the silicon sulfides produced by a reaction between Si and sulfur act as both the source gas and the melted catalyst. In contrast, the growth of oxygen-rich nanowires is dominant at higher temperatures. There is a very small amount of liquid SiS_2_ acting as a melted catalyst at very high temperatures because of the melting point of SiS_2_ at 1090 °C. Therefore, the epitaxial growth of Si nanowires by the metal-catalyst-free process diminishes at higher temperatures, and bent nanowires with lumps are grown through oxide-assisted growth.

## Methods

The growth of epitaxial Si nanowires by a metal-catalyst-free process was performed using a simple method. First, the Si substrates were cut from a (111)-oriented Czochralski (CZ) Si wafer. Chemical etching of the Si substrate surface was performed in 5% hydrofluoric acid to remove the silicon oxide. Next, sulfur powder (99.99%) and the Si substrate were sealed in a quartz capsule that was evacuated to a base pressure of approximately 20 Pa. The quartz capsule was transferred into a horizontal furnace and annealed at different temperatures between 1000 and 1300 °C for 30 min. The quartz capsule was then quenched in water after the furnace was turned off. For reference, we also prepared an Au-covered Si substrate. In this case, a 5-nm-thick Au layer was deposited on Si substrates by vacuum thermal evaporation. Sulfur powder (99.99%) and the Au-covered Si substrate were sealed in a quartz capsule and annealed at approximately 1200 °C for 30 min. The morphologies of the as-grown samples were obtained by scanning electron microscopy (SEM; JEOL JSM-6300) and field emission-SEM (FE-SEM; Hitachi SU8000). The crystallinity of the nanowire was confirmed by transmission electron microscopy (TEM; JEOL JEM-2100F). The elemental compositions of the nanowires were estimated by energy dispersive X-ray fluorescence (EDX).

## Additional Information

**How to cite this article**: Ishiyama, T. *et al*. Growth of epitaxial silicon nanowires on a Si substrate by a metal-catalyst-free process. *Sci. Rep.* **6**, 30608; doi: 10.1038/srep30608 (2016).

## Supplementary Material

Supplementary Information

## Figures and Tables

**Table 1:** List of the synthetic conditions used to prepare Si nanowires by various methods without any metal catalysts.

	Method	T (°C)	Pressure (Pa)	Precursor	Ref.
Oxygen-assisted growth	Laser ablation	930	Ar at 7 × 10^4^	SiO	27, 28
		850–1050	Ar atmosphere	SiO	29
		1130–1400	Ar atmosphere	SiO	30
	CVD	900–1000	Ar at 3 × 10^4^	SiO	31
		490	2 × 10^3^	SiH_4_	35
	SFC	430–550	1 × 10^7^	Phenylsilane	36
Carbothermal growth	CVD	1200–1350	Ar atmosphere	SiO	33
		950–1100	Ar atmosphere	SiO	34
Sulfur-assisted growth	CVD	1200	20	silicon sulfide	This work
