# Supplementary material for: Growth of epitaxial silicon nanowires on a Si substrate by a metal-catalyst-free process
Source: Sci Rep. 2016 Jul 28;6:30608. doi: 10.1038/srep30608 (PMC4964331; doi:10.1038/srep30608)
Supplement: Supplementary Information [file srep30608-s1.pdf]

Growth of epitaxial silicon nanowires on a Si substrate by a metal-catalyst-free process

Takeshi Ishiyama*, Shuhei Nakagawa, and Toshiki Wakamatsu
Department of Electrical and Electronic Information Engineering, Toyohashi University of Technology, 1-1 Hibarigaoka, Tempaku-cho, Toyohashi, Aichi 441-8580, Japan

*E-mail:address: ishiyama@ee.tut.ac.jp

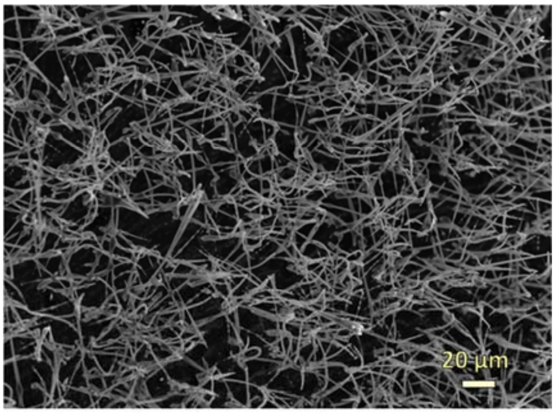

Fig. S1. SEM image of nanowires grown at 30 Pa. The growth of bent nanowires with high oxygen contents is more dominant because residual oxygen in the quartz capsule is incorporated into nanowires.
